# Fatal Interstitial Pneumonia Associated with Bovine Coronavirus in Cows from Southern Italy

**DOI:** 10.3390/v12111331

**Published:** 2020-11-19

**Authors:** Maria Grazia Amoroso, Giuseppe Lucifora, Barbara Degli Uberti, Francesco Serra, Giovanna De Luca, Giorgia Borriello, Alessandro De Domenico, Sergio Brandi, Maria Concetta Cuomo, Francesca Bove, Marita Georgia Riccardi, Giorgio Galiero, Giovanna Fusco

**Affiliations:** 1 Unit of Virology, Department of Animal Health, Experimental Zooprophylactic Institute of Southern Italy, Via Salute 2, 80055 Portici, Italy; barbara.degliuberti@cert.izsmportici.it (B.D.U.); francesco.serra@izsmportici.it (F.S.); giovanna.deluca@izsmportici.it (G.D.L.); sergio.brandi@cert.izsmportici.it (S.B.); giovanna.fusco@cert.izsmportici.it (G.F.); 2 Section of Vibo Valentia, Experimental Zooprophylactic Institute of Southern Italy, Contrada Piano di Bruno, 89852 Mileto, Italy; giuseppe.lucifora@cert.izsmportici.it; 3 Department of Animal Health, Experimental Zooprophylactic Institute of Southern Italy, Via Salute 2, 80055 Portici, Italy; giorgia.borriello@izsmportici.it (G.B.); marycc@live.it (M.C.C.); francesca.bove@izsmportici.it (F.B.); maritageorgia.riccardi@izsmportici.it (M.G.R.); giorgio.galiero@cert.izsmportici.it (G.G.); 4 Freelance Veterinary, Ordine dei Veterinari di Vibo Valentia, 89900 Vibo Valentia, Italy; alessandro.dedomenico@yahoo.com

**Keywords:** bovine coronavirus, interstitial pneumonia, phylogenetic analysis, real time PCR

## Abstract

An outbreak of winter dysentery, complicated by severe respiratory syndrome, occurred in January 2020 in a high production dairy cow herd located in a hilly area of the Calabria region. Of the 52 animals belonging to the farm, 5 (9.6%) died with severe respiratory distress, death occurring 3–4 days after the appearance of the respiratory signs (caught and gasping breath). Microbiological analysis revealed absence of pathogenic bacteria whilst Real-time PCR identified the presence of RNA from Bovine Coronavirus (BCoV) in several organs: lungs, small intestine (jejunum), mediastinal lymph nodes, liver and placenta. BCoV was therefore hypothesized to play a role in the lethal pulmonary infection. Like the other CoVs, BCoV is able to cause different syndromes. Its role in calf diarrhea and in mild respiratory disease is well known: we report instead the involvement of this virus in a severe and fatal respiratory disorder, with symptoms and disease evolution resembling those of Severe Acute Respiratory Syndromes (SARS).

## 1. Introduction

Coronaviruses (CoVs) are enveloped viruses, with a RNA single-stranded large genome of 27.6–31 kb [1]. This big group of viruses, belonging to the order *Nidovirales,* suborder *Coronavirinae*, family *Coronaviridae*, subfamily *Orthocornavirinae*, is divided, (on the basis of the antigenic and genetic properties of these viruses [1,2] into 4 genera: alpha, beta, gamma and delta coronavirus. Coronaviruses are responsible for enteric, respiratory and neurological diseases in both birds and mammals, including humans [3]. Bats and birds are major reservoir of CoVs and some CoVs are endemic in domestic animals in different countries [4]. Several coronaviruses are well known for their ability to change tissue tropism, to pass species barriers and to adapt to new ecological niches. These abilities are related to two factors commonly occurring during RNA replication: accumulation of point mutations (one mutation for each round of replication) caused by the low fidelity of their RNA dependent RNA polymerase (RdRp), as well as homologous and heterologous recombination events [1,2]. These features promote the emergence of novel strains with novel biological properties such as host range, tissue tropism and virulence [1,5]. The high mutation and recombination rates are indeed implicated in the emergence of new human coronaviruses, of animal origin, as occurred for the appearance of the strains HCoV-229E and HCoV-OC43 (the only two human coronaviruses known before SARS emergence), as well as for the arising of SARS-CoV Middle East Respiratory Syndrome Coronavirus Infection, (MERS-CoV) and the recent global pandemic SARS-CoV-2 [1,3,6,7,8]. CoVs infections in veterinary medicine are well known from a long time [1]. The most common animal CoVs are: infectious bronchitis virus (chickens), porcine transmissible gastroenteritis, porcine hemagglutinating encephalomyelitis and porcine epidemic diarrhea CoVs (swine), bovine coronavirus (BCoV, cattle), canine enteric and canine respiratory CoVs (dogs), and feline coronavirus (cats) [3]. BCoV belongs, together with human coronavirus (OC43), human enteric coronavirus (4408) and canine respiratory coronavirus (CRCoV), to subgroup A of the betacoronavirus genus [9,10,11]. This virus is pneumoenteric and has a dual tissue tropism, infecting both intestine and respiratory apparatus (upper and lower tracts), a feature in common with human SARS and SARS-CoV-2 [5]. It is endemic worldwide and is considered one of the major causes of neonatal calf diarrhea, with mortality related to virus ability to destroy the intestinal villi and to cause severe bloody diarrhea [2]. In adult dairy cattle, BCoV is instead associated with the well-known winter dysentery (WD), which causes severe drop in milk production and consequent significant economic losses for the herds [2]. In cattle of various ages, the virus is also responsible for respiratory infections (bovine respiratory disease complex, shipping fever) of feedlot cattle [5]. In our study we report the unusual course of a clinically diagnosed WD in a dairy cattle herd of Southern Italy, in which the disease has never been described before. The majority of affected animals showed gastrointestinal disease with bloody diarrhea and mild respiratory symptoms (cough, slight temperature increase, nasal discharge). Among them, 5 displayed a severe respiratory illness, which evolved into death within few days after the occurrence of the first respiratory difficulties. To ascertain the cause of death, animals underwent necroscopy and organs were analyzed to investigate the presence of possible bacterial and viral pathogens respectively by cultural and molecular methods.

## 2. Materials and Methods

### 2.1. Outbreak Description

The herd consisted of a total population of 52 Italian Friesian cows, 29 of which in lactation. Cattle were housed under a single canopy and separated into production groups with feeders on the central corridor and drinking bowls inside each paddock. The production system was characterized by internal recovery. No movements or purchases occurred in the three years before the occurrence of the WD outbreak. At the beginning of January 2020 most of the lactating cows, all vaccinated against bovine alpha-herpesvirus 1 (BoHV-1) and bovine viral diarrhea virus (BVD), began to experience symptoms of disease clinically diagnosed as WD. In details, 25 lactating cows (86%) showed bloody diarrhea and a decrease in milk production up to two thirds. Dry cows and juveniles showed no symptoms. After about 2days, the majority of cows with diarrhea exhibited respiratory symptoms, with cough and catarrhal secretion, dyspnea and moderate febrile rise (rectal temperature, 39.2–40.5 °C). In 5 of them, the symptoms worsened with manifestation of subcutaneous emphysema and pneumothorax. In particular two four months pregnant lactating cows (about 4 years old) began to show mild respiratory symptoms which rapidly aggravated becoming severe and one died in 24 h. The other one died the next day. Initially, only symptomatic treatments (ensure clean water and add dry feed) were prescribed but, after the death of the first cow, antibiotics and corticosteroids were administered to the other animals. Despite the pharmaceutical treatments, 3 more animals, (about 7 years old), died after the appearance of the same respiratory symptoms. About ten days later, respiratory and gastrointestinal symptoms of the other animals started to regress. Dead animals underwent necropsy to investigate the cause of death and organs, together with fecal material, were analyzed for the presence of bacterial, viral and parasite pathogens.

### 2.2. Histopathological Analysis

Tissues of the organs were fixed in 10% neutral phosphate-buffered formalin and processed by routine methods into paraffin blocks which were cut into 3–4 μm thick sections and stained with hematoxylin and eosin.

### 2.3. Bacteriological and Parasitological Analysis

Lungs, liver, kidneys, spleen, meseraic and supramammary lymphnodes as well as cephalorachid fluid of the first two dead cows were tested by bacterial culture methods to investigate the presence of bacterial pathogens possibly associated with the disease and the death. Briefly, samples were inoculated on both MacConkey agar no. 3 (CM0115 Oxoid, Thermo Fisher Scientific, Waltham, MA, USA) plates and blood agar (7% *v*/*v* ovine blood in blood agar base, CM0271 Oxoid, Thermo Fisher Scientific, Waltham, MA, USA) plates. MacConkey agar plates were incubated at 37 °C for 48 h, while blood agar plates were incubated at 37 °C for up to 72 h in presence/absence of 5% CO_2_. The presence of *Mycoplasma* spp. was investigated in lungs and kidneys following OIE terrestrial manual 2018 (Chapter 3.4.8) protocol. In brief: samples were grinded in broth and inoculated both undiluted onto five solid media plates (*Mycoplasma* agar) and diluted 1:10 into five tubesof broth (*Mycoplasma* broth). Plates and tubes were incubated at 37 °C in a 5%CO_2_ atmosphere and were inspected daily for 21 days. All the suspected colonies, isolated by microbiological methods as above described, underwent species confirmation by taxonomic identification through MicroSEQ^®^ rapid microbial identification system (Thermo Fisher Scientific, Waltham, MA, USA), which is based on the 16s rRNA gene sequencing.

Fecal material collected during the disease state was analyzed for the presence of helminth eggs and coccidia oocysts by routine methods.

### 2.4. Viral Nucleic Acids Extraction Procedures

Samples (25 mg of tissue in 1 mL Phosphate buffered saline solution) from lungs, liver, kidneys, spleen, mediastinal lymphnodes, placenta, foetus and intestine content of the two animals were homogenated by Tissue Lyser (Qiagen GmbH—Hilden, Germany). Each organ was analyzed in triplicate, by sampling three different parts of the whole tissue, each one in turn analyzed in triplicate. In particular for the lungs we analyzed three samples from the left cranial lobe. Nucleic acid extraction was carried out from 200 µL of each organ homogenate by using QIAsymphony automated extraction system (Qiagen GmbH, Hilden, Germany) with the DSP Virus/Pathogen Mini kit (Qiagen GmbH, Hilden, Germany) according to the manufacturer’s instructions. Nucleic acids were eluted in 60 µL of elution buffer containing 40 unit/µL RNase inhibitor (Promega Corporation, Madison, Wisconsin, USA) and immediately analyzed by Real-time PCR.

### 2.5. Real Time Virus Identification

DNA viruses like bovine alphaherpesvirus 1 (BoHV-1), bovine alphaherpesvirus 2 (BoHV2), bovine alphaherpesvirus 4 (BoHV4) were investigated by Real-time polymerase chain reaction using Quantitect Real time PCR detection kit (Qiagen GmbH). RNA viruses: bovine coronavirus, (BCoV), bovine viral diarrhea virus (BVDV), border disease virus (BDV), parainfluenza virus 3 (PI3) and respiratory syncytial virus (RSV) were analyzed by Real-time reverse transcription polymerase chain reaction using AGPATH reaction kit (Thermo Fisher Scientific). All the reactions were carried out with primers (Tema Ricerca srl, Castenaso, Bologna, Italy) and probes (Thermo Fisher Scientific) specific for the tested virus on a Quantstudio5 system (Thermo Fisher Scientific) with protocols routinely used in our laboratory. In particular BCoV was identified as described in literature [12], and BCoV positive control was kindly given by Friederich institute (Greifswald, Insel Riems, Germany). Organs were considered positive when at least one sample exhibited a mean threshold cycle (C_t_) < 40. Threshold cycle of the positive organ was calculated as an average of the 3 replicates analyzed.

### 2.6. BCoV Sequencing and Strain Characterization

All the samples positive for bovine coronavirus, as well as the strain isolated by tissue culture were further processed by sequencing analysis to characterize the identified strains. In details, 5 uL of nucleic acids were amplified by a RT-PCR reaction targeting the partial sequence of two genes: a 456 bp fragment of the RNA-dependent RNA polymerase (*RdRp*) gene and a 454 bp fragment of the Nucleocapsid protein gene (*N*). In details RdRp PCR reaction was carried out as indicated in literature [13] with AGPATH reaction kit (Thermo Fisher Scientific), using 1.25 µL of each primer (10 µM) with the following thermal profile: 50 °C for 30 min, 95 °C for 15 min, 45 cycles of 94 °C for 30 s, 50 °C for 30 s, 68 °C for 30 s and a final elongation step of 72 °C for 10 min. RT-PCR reaction for the *N* gene was carried out as reported [14] with 5 uL of nucleic acids using the AGPATH reaction kit (Thermo Fisher Scientific) and 2 µL of each primer (10 µM).The thermal profile was: 42 °C for 30 min, 95 °C for 10 min, 40 cycles of 94 °C for 1 min, 55 °C for 1 min, 72 °C for 1 min and a final elongation step of 72 °C for 7 min. PCR products were analyzed by Tape station (Agilent Technologies—Santa Clara, CA, USA) using the D 1000 kit. Amplicons were sequenced by capillary electrophoresis as previously described [15]. The nucleotide sequence similarity searches were performed using the BLAST server (http://www.ncbi.nlm.nih.gov/genbank/index.html) and phylogenetic analysis was carried out using the Jalview vs. 2.10.5 (www.jalview.org) and MEGA vs. 7.0.26 (www.megasoftware.net) softwares.

### 2.7. Virus Isolation

Virus isolation was attemptedinHRT18 cells, Vero cells and A72 cells. HRT-18 cells were grown in Dulbecco’s modified Eagle’s Medium (D-MEM, Gibco, Thermo Fisher Scientific, Waltham, MA, USA), supplemented with 10% fetal bovine serum (FBS, Gibco, Thermo Fisher Scientific, Waltham, MA, USA), 1% antibiotic/antimicotic (Gibco, Thermo Fisher Scientific, Waltham, MA, USA), 1% L-glutamine (Gibco, Thermo Fisher Scientific, Waltham, MA, USA). Vero cells and A72 cells were cultured as above indicated but in MEM. BCoV isolation was carried out in cells cultured in 2% FBS medium. One ml of each organ (positive to BCoV) homogenate was diluted 1:10, filtered and inoculated in cell monolayers. After one hour of adsorption at 37 °C the inoculum was discarded. Cells were incubated at 37 °C and cytopathic effect was daily observed until 5 days after inoculation.

## 3. Results

### 3.1. Necroscopy Findings

**Figure 1:**
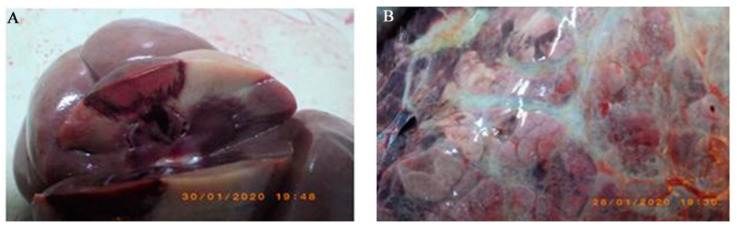
(**A**) Kidney showing infarcts involving cortical and medullary tissue. (**B**) Lung with diffuse fibrinous pleurisy and emphysematous interstitial pneumonia.

Of the 52 cows present in the breeding farm, 5 (9.6% of the animals, 17.2% of the lactating cows) died with severe respiratory distress, death occurring 1–4 days after the appearance of the respiratory signs (caught and gasping breath). Anatomo-pathological inspection of the dead cows showed similar lesions in all animals as herein described. We first observed congested explorable mucous membranes and hemorrhagic lymphadenitis of the supra-mammary glands. Jejunum was affected by hemorrhagic enteritis with the rectal ampoule containing little hemorrhagic diarrhoea. There was catarrhal lymphadenitis of the mesenterics. Liver showed increased volume and was characterized by sub-capsular hemorrhages and diffuse centro-lobular necrosis. Moderate splenomegaly with sub capsular extravasations at the visceral surface was observed. Kidneys showed red infarcts involving cortical and medullar tissues (Figure 1A). Foetus of around 4 months old was characterized by diffuse subcutaneous hemorrhages. With respect to thoracic cavity we observed diffuse fibrinous-hemorrhagic pleurisy; thymus was studded with hemorrhages. Respiratory apparatus showed severe tracheo-bronchitis and muco-hemorrhagic bronchiolitis with the deposition of fibrin casts. Lungs (Figure 1B) were characterized by diffuse fibrinous pleurisy, emphysematous interstitial pneumonia and hemorrhagic mediastinal lymphadenitis. The heart, in diastole, revealed hemorrhages of the epicardium along the coronary and ventricular endocardium, thinning of the right ventricular wall and thickening of the left, with heart basis showing fibrinous epicarditis.

### 3.2. Histopathology

**Figure 2:**
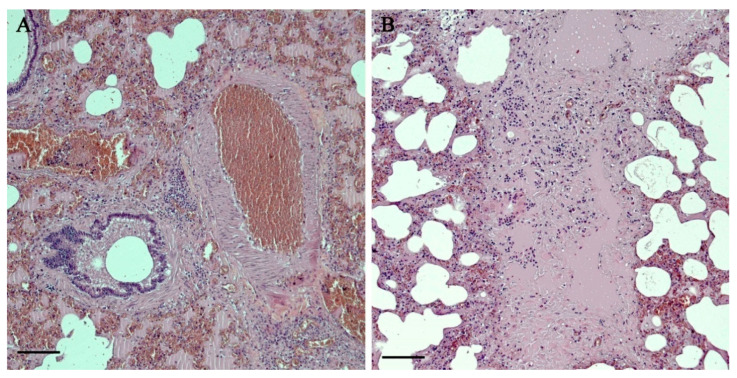
Histopathological observation of the lungs (10× magnification field). (**A**): vascular thrombosis, lympho-plasmacellular vasculitis and oedema. (**B**): interstitial pneumonia, oedema, fibrosis.

Microscopic examination of the lungs (Figure 2A,B) demonstrated pleural thickening and fibrin mixed with extra-pleural exudates with prevalent inflammatory population of neutrophils. It was observed the presence of areas of sub-pleural emphysema and alveolar rupture with accumulation of dense eosinophilic material as well as multifocal inflammatory thickening of the pulmonary interstitium with a clear prevalence of foamy macrophages, lymphocytes and plasma cells. Lympho-plasmacellular vasculitis and thrombosis were also described. The bronchial and bronchiolar lumen showed accumulation of dense material and cellular debris mixed with macrophages and lymphocytes. Moderate hyperplasia of the bronchiole-associated lymphoid tissue (BALT) was visible.

**Figure 3:**
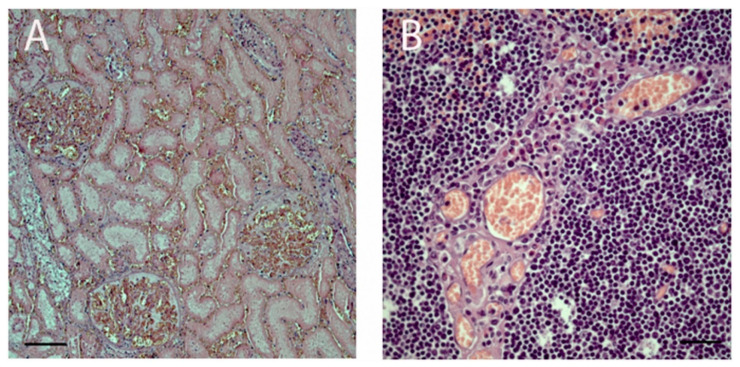
Histopathological observation of (**A**): kidney (10× magnification field) with glomerular thrombosis, tubular ischemic necrosis and interstitial congestion; (**B**) thymus (40× magnification field) characterized by vasculitis and ischemic necrosis.

Sporadic multinucleated syncytial cells in the peri-bronchial site were identified. Ischemic necrosis of a thrombotic nature from vasculitis, characterized by the presence of fibrin, lymphocytes, plasma cells and rare granulocytes, was found in the liver, spleen, kidney (Figure 3A), thymus (Figure 3B) and skin of the fetus. Jejunum presented tissue disruption and morpho-structural alteration of the villi. Lymphocytes and granulocytes with a clear prevalence of eosinophils were mainly represented to mediate the inflammatory process into lamina propria and interstitium of mucosa.

### 3.3. Microbiology and Virology

Microbiological analysis revealed absence of pathogenic bacteria in all the analyzed organs. No parasites were recognized in the fecal material. Real time PCR carried out on the organs of the animals gave negative results for all the herpesviruses investigated as well for: BVDV, BDV and PI3. The only pathogen identified was BCoV, that was detected in: lungs, small intestine (jejunum), mediastinal lymph nodes, liver and placenta. Foetal thymus, kidney and spleen were negative for the virus. Among the positive organs, lungs showed a very high signal of fluorescence in both the cows analyzed, with a mean threshold cycle (C_t_) of 19.8 and 20.2, respectively. The other organs displayed instead a mean C_t_ value ranging from 25 to 32. Virus was successfully isolated from lungs and jejunum on both A72 and Vero cells. Cells showed cytopatic effect after 4 days of culturing and isolates were considered positive when presenting the effect until the fourth serial passage. Isolated strains were confirmed as BCoV by both Real time PCR and sequencing.

### 3.4. Sequencing Analysis of BCoV

**Figure 4:**
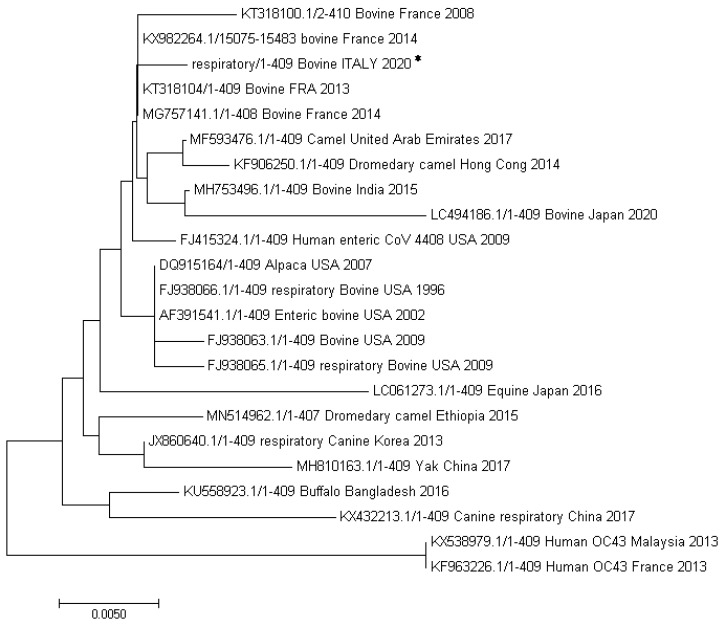
Analysis of a part of the *RdRp* gene (409 bp). The evolutionary history was inferred using the Neighbor-Joining method [16]. The optimal tree with the sum of branch length = 0.10942734 is shown. The evolutionary distances were computed using the Poisson correction method and are in the units of the number of amino acid substitutions per site. Evolutionary analyses were conducted in MEGA7 [17]. Sequence obtained in the present study (Bovine ITALY 2020) is indicated with an asterisk.

All the bovine coronavirus sequences directly obtained from the positive organs (lungs, jejunum, lymphnodes, liver and placenta) as well as those obtained from the virus isolated in cell culture were aligned and compared by Jaleview analysis software. All the sequences resulted identical to each other with respect to the two gene fragments investigated. Further phylogenetic analysis of the BCoV identified (partial *RdRp* gene, Genbank accession number, AC: MT602514) revealed (Figure 4), the highest homology (99%) with only one base substitution (C instead of A), with two bovine strains isolated in France from diarrheic fecal samples in 2013 (AC: KT318104) and 2014 (AC: KX982264), respectively. Among the bovine coronaviruses, our strain was also closely related (two nucleotides of difference) to a BCoV identified from a nasal swab pool in France in 2014 (MG757141.1), a BCoV isolated in India in 2015 from feces, and two BCoVs (one respiratory and one enteric) isolated in USA in 1996 and 2002, respectively. Among the non-bovine CoVs, the highest homology was shown with a bovine-like CoV isolated from an alpaca in USA in 2007, with a CoV isolated in a camel in the United Arab Emirates in 2017 and with a human enteric coronavirus 4408 (FJ415324) collected in 1988 from a patient in Germany.

**Figure 5:**
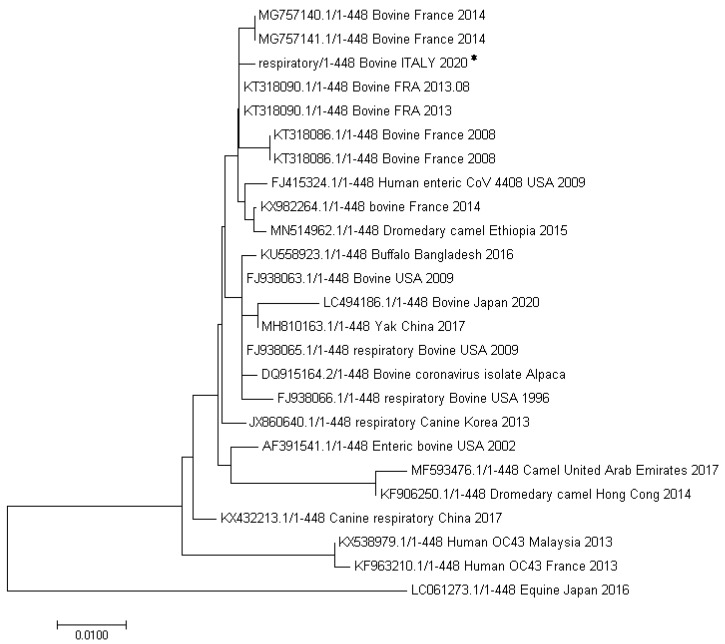
Phylogenetic analysis of a part of the *N* gene (454 bp). The evolutionary history was inferred using the Neighbor-Joining method [16]. The optimal tree with the sum of branch length = 0.10942734 is shown. The evolutionary distances were computed using the Poisson correction method and are in the units of the number of amino acid substitutions per site. Evolutionary analyses were conducted in MEGA7 [17]. Sequence obtained in the present study (Bovine ITALY 2020) is indicated with an asterisk.

With respect to gene N (Figure 5), our sequence (GenBank accession MW074864) showed the highest homology (99%), with an enteric strain isolated from a bovine in France in 2013 (AC: KT318090.1), with only one base substitution (A instead of C).Among the bovine coronaviruses, our BCoV was also closely related (two nucleotides of difference) to two BCoVs identified in cows in France in 2014 respectively from deep nasal swabs and from bronchoalveolar lavages [18]. These samples were collected from herds affected with respiratory disease in the south-east of France from calves with acute signs of pneumonia. Among the non-bovine CoVs, the highest homology was shown with a dromedary camel CoV isolated in Ethiopia in 2015 and, like for the *RdRp* gene, with a human enteric coronavirus 4408 (FJ415324) collected in 1988 from a patient in Germany from which our sequence differs for 3 nucleotides.

## 4. Discussion

In our study we described a severe pulmonary infection, causing death in 5 animals from a high genealogy Friesian dairy cows breeding. The infection involved 48% of the animals (almost all the lactating cows), mostly exhibiting gastrointestinal and mild respiratory symptoms. Among infected animals, 20% (5 out of 25) experienced a stronger form of the disease, leading to death in a few days after the beginning of respiratory symptoms. In the samples analyzed we failed to identify any bacteria and virus typically responsible for pneumonia in bovines. The only pathogen found was BCoV. For this reason, the involvement of this virus in the fatal pneumonia was hypothesized, even though it was not possible to definitively conclude whether it was the primary cause of the disease or represented a secondary infection aggravating the symptoms.

With respect to BCoV role in the pneumonia herein described, as a matter of fact while the involvement of BCoV in enteric infections is well known, its role in bovine respiratory disease is controversial and principally related to mild respiratory symptoms. The virus has been indeed found in respiratory samples from both healthy and sick cattle [11,19,20,21,22,23] and more frequently identified in association with other respiratory pathogens [11,20,24,25,26]. Only few papers reported instead BCoVas the only recognized cause of the disease [9,24,27]. Furthermore, there are various studies in literature describing attempts to induce clinical respiratory symptoms by infecting healthy cattle with BCoV. Most experiments reported no respiratory symptoms development, with, in some cases the occurrence of gastrointestinal disease [28,29,30,31], while only few studies describe slight respiratory problems [32,33]. Our results seem to corroborate the role of this virus in respiratory outbreaks and to point out the ability of the virus to induce, in the same animal or within the same outbreak, both enteric and respiratory symptoms, confirming what already reported by Choulienko et al. [34]. With respect to the 5 fatal pneumonia cases, the observed exacerbation of the respiratory disease in these animals, resembling what happens for other severe coronavirus syndromes (like those caused by SARS, MERS, COVID-19), could be related to various co-factors including: immunosuppression (due to corticosteroids treatment), individual host susceptibility, co-morbidities, physical stresses [5]. Moreover, since through recombination events CoVs are able to gain novel biological properties, including an increased pathogenicity [1] we could also hypothesize the identification of a more pathogenic variant of bovine coronavirus, with acquired ability to cause fatal pneumonia in susceptible hosts. Interestingly, among the BCoV nucleotide sequence data available in GenBank to which our strain was compared, we observed close similarity, (with respect to *RdRp* and *N* genes) to sequences obtained from cows in France in 2014 [18]. These animals all exhibited acute signs of pneumonia but for all of them it was described a co-infecting bacterial or viral agent. In our case we failed to detect any other pathogen and could thus address the disease only to BCoV. With respect to bacteria, isolation can be compromised by antibiotic treatments which are usually prescribed too fast (at first respiratory symptoms appearance) by veterinarians. In our case, since the farm veterinary diagnosed the outbreak as WD, animals were not treated with antibiotics until the first death. We cannot completely exclude however that the breeder was autonomously already giving drugs to the symptomatic cows. Further studies including full genome sequencing and experimental infections should be done to better characterize the strain of BCoV and to shed light on its likely real ability to cause severe pneumonia like the one we herein described.

Many bovine-like CoVs have been indeed identified as enteric and/or respiratory pathogens in both livestock and wildlife species (wild ruminants, captive ruminants as well as water buffalo, camelids) [5,10,35,36,37,38,39]. With respect to the possibility of BCoV-like viruses to infect humans, in literature it has been described a case of child acute enteritis caused by a human CoV found genetically and antigenetically more closely related to BCoV than to HCoV-OC43 [40]. Moreover HCoV-OC43 has been supposed to derive from an ancestral BCoV strain (considering their close genetic and antigenic similarity) which skipped the species barrier and passed from rodents to humans through cattle [41,42,43]. Interestingly, phylogenetic analysis showed that our strain, with respect to the analyzed part of *RdRp* gene, was very closely related to an enteric HCoV-4408 described in a patient in Germany (see Figure 4 and Figure 5). Interspecies transmission via wildlife and livestock host animals are key factors for the emerging of new, highly pathogenic, human coronaviruses. For this reason, it is of utmost importance to focus on and carefully investigate the outbreaks in which animal coronaviruses are involved, with particular regard to clinical symptoms and genetic classification. Finally, in the case of BCoVs, it is necessary to recommend the farmers to strictly follow all the protection and prevention biosafety measures necessary to limit the spread of such viruses among animals and to avoid any possible human contamination.
